# Universal Study Design for Instrument Changes in Pharmaceutical Release Analytics

**DOI:** 10.1002/elps.70004

**Published:** 2025-06-25

**Authors:** Anne B. Ries, Maximilian N. Merkel, Kristina Coßmann, Marina Paul, Robin Grunwald, Daniel Klemmer, Franziska Hübner, Sabine Eggensperger, Frederik T. Weiß

**Affiliations:** ^1^ Quality Control Boehringer Ingelheim Pharma GmbH & Co. KG Biberach an der Riss Germany; ^2^ CMC Statistics BioPharma Boehringer Ingelheim Pharma GmbH & Co. KG Biberach an der Riss Germany

**Keywords:** comparability | instrument update | instrument replacement | ICE3 to Maurice | quality control | state-of-the-art

## Abstract

In pharmaceutical quality control (QC), analytical methods need to maintain release analytics over decades. Over a product's lifecycle, vendors may update instrument hardware and/or software, or a switch between vendors may become necessary upon discontinuation of an instrument. Both situations pose a challenge to pharmaceutical QC.We designed an efficient instrument comparability study to gain a comprehensive understanding of potential performance differences between instruments and therefore rationalize the risk assessment and decision process for a path forward. The results may either point out whether a full or partial re‐validation is necessary or whether a science‐based update can be pursued based on the data generated in the study. The study design is universally applicable to a substantial range of release analytical methods. In a straightforward setup of two experiments with the new instrument, a statistically meaningful data set is generated for comparison with available historical or validation data of the original instrument. In a Good Manufacturing Practice (GMP) environment, we implemented the study design in a benchmark study comparing the ICE3 and Maurice C imaged capillary isoelectric focusing (icIEF) instruments. The core‐study confirmed equal or better performance of the Maurice C in all parameters and serves as a basis for seamless continuation of release icIEF measurements on Maurice C.

**Abbreviations**
DoE: design of experiments
GMP: good manufacturing practice
icIEF: imaged capillary isoelectric focusing
QC: quality control
SST: system suitability test
TOST: two one‐sided *t*‐tests

## 1 Introduction

Analytical methods in pharmaceutical quality control (QC) need to be maintained for decades to guarantee release analytics and ongoing process verification over the life cycle of commercial products [1, 2, 3, 4].

If, for example, a vendor updates hardware or software on an analytical instrument, then product specific analytical procedures validated on the original equipment must be moved to the new equipment [2]. The implementation of such technical changes in a regulated environment typically poses a challenge and is of high interest in the pharmaceutical field [5, 6, 7].

Depending on the impact of the technical change on the analytical result, a science‐based instrument update, or a partial/full method re‐validation may be considered [2, 8, 9, 10, 11]. The impact also determines the type of change and regulatory paths to follow when implementing the new instrument [12].

In line with ICH Q2(R2), Q9(R1), and Q14 [9, 10, 11], we developed a universal study design to assess instrument comparability efficiently and comprehensively, to minimize subjectivity, and to provide a decision in the path‐forward of instrument modernization in a good manufacturing practice (GMP) environment. If instrument comparability can be fully confirmed, the study design may well serve as scientific justification to seamlessly continue release analytics on the new instrument (science‐based update).

The proposed study design aims at separation methods, such as capillary‐electrophoretic or chromatographic methods, which produce a product profile in a graph readout, but the underlying concept can be adapted to other methods. Two experiments on the new instrument are sufficient to generate statistically meaningful data. For evaluation of comparability with the original instrument, no further experiments are required to be carried out. Instead, validation and historical data are used.

If multiple products are affected by the instrument change, the product with the highest complexity in readout (e.g., close peaks, high number of peaks) and sample preparation (e.g., high number of incubations, buffer exchanges) is used in the comparability study. Thereby, the study results may be considered indicative for further affected products/procedures.

As a benchmark, we applied the study design in a comparability assessment of two imaged capillary isoelectric focusing (icIEF) instrument platforms ICE3 (Protein Simple) and Maurice C (Protein Simple) in a GMP environment.

## 2 Theory

In an ideal situation, two unique instrument platforms of the same analytical purpose produce identical results. In a real world situation, discrepancies occur, and it is key to compare the instrument readouts to understand their extent.

**FIGURE 1:**
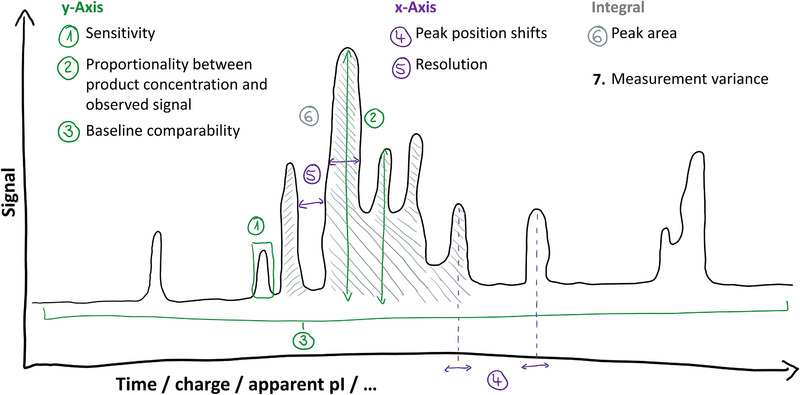
Visualization of parameters to be assessed in *y*‐dimension (green labels 1–3), *x*‐dimension (purple labels 4 and 5), and the curve integral (grey label 6) of the profile. The 7. assessed parameter is the variance between measurements (black label 7, not visualized in the drawing).

To systematically assess comparability of graph readouts, such as electropherograms or chromatograms, we considered discrepancies along the *x*‐axis, *y*‐axis, and within the curve integral (Figure 1). Furthermore, we took the method variance between measurements into account. These considerations led to the identification of seven parameters:

- *y*‐axis
  1. sensitivity
  2. proportionality between product concentration and observed signal
  3. baseline comparability

- *x*‐axis
  4. peak position shifts
  5. resolution

- integral
  6. peak area

- measurement variance
  7. across different instruments, analysts, days, capillaries/columns.

Acceptance criterion for instrument comparability is the observation of equal or better performance of the new instrument [2]. Individual acceptance criteria for each parameter may be set from historical data such as method validation or control chart data from the original instrument. Visual comparison may be advisable for some parameters. Each parameter and respective criteria are described in the following sections.

### 2.1 Sensitivity

To evaluate the comparability of detection sensitivity and signal intensities between two instrument platforms, the signal‐to‐noise (S/N) ratio of a robust peak is used. A small peak, such as the limit of quantitation (LOQ) peak from original method validation, is typically well suited with historical S/N data likely available. S/N values on the new instrument can be determined from the same peak in a sufficient amount (e.g., same number of data points as used for LOQ determination during validation).

Robustness of the S/N peak should be verified, for example, with the peak area relative standard deviation (RSD), which is also for the LOQ peak likely available from method validation on the original instrument.

The LOQ value itself is an area parameter and should not be considered for sensitivity evaluation, as peak resolution may impact the LOQ value. The S/N value is a pure *y*‐axis parameter and therefore more accurate in sensitivity comparison.

#### 2.1.1 Statistical Evaluation

Non‐inferiority of the S/N values on the new instrument compared with the mean of S/N values from validation on the old instrument may be assessed with a one‐sided *t*‐test (*α* = 0.05).

The S/N peak RSD point value calculated from measurements on the new instrument should be identical or lower than the RSD point value available from the original instrument.

### 2.2 Proportionality Between Product Concentration and Observed Signal

To evaluate if sample concentration and detected signal intensity show a comparable proportionality on the new instrument, a response experiment needs to be performed, following the procedure and acceptance criteria of the original method validation on the original instrument.

#### 2.2.1 Statistical Evaluation

The acceptance criteria for response in the original validation should be met by the response analysis on the new instrument. Typical criteria may include a limit in correlation coefficient *r* over the linearity range (e.g., *r* ≥ 0.98) or in the coefficient of determination *R* ^2^.

In addition to the validation criteria, the validation results may be compared to the results acquired on the new instrument.

### 2.3 Baseline Comparability

Profile baselines need to be compared between both instruments to investigate potential trends in the *y*‐dimension of the graph. The parameter can be evaluated through comparison of blank measurements. Multiple (e.g., three) representative blank measurements from historical data of the original instrument and data acquired on the new instrument may be compared in a direct overlay.

#### 2.3.1 Evaluation

Visual comparison allows identification of potential irregularities or discrepancies in putative method specific blank patterns. Irregularities in the blank measurements of the new instrument should be similar or less pronounced than on the original instrument.

### 2.4 Peak Position Shifts in *X*

To evaluate consistency of the peak positions in the *x*‐dimension of the graph, exact *x*‐values of multiple (e.g., three, distributed over the separation range of the product) specific peaks should be determined. The peak position data should consist of at least 12 independent data points collected from a matrix setup (Section 3.1; Kojima design [13], Table S1).

#### 2.4.1 Statistical Evaluation

For the peak positions on both instruments to be consistent, the acquired data from the new instrument should be in a range of ±3 standard deviations (SD) of the historical data acquired on the original instrument (e.g., control chart data). The comparability of both instruments should be statistically evaluated with a two one‐sided *t*‐test (TOST), using a significance threshold of *α* = 0.05.

### 2.5 Resolution

To assess graph resolution, peak widths and peak separation on both instruments need to be compared.

Therefore, multiple (e.g., three) representative sample measurements from historical data and data acquired on the new instrument may be visually compared in a direct overlay.

In case the selected product/analytical procedure provides baseline‐separated data, resolution should be determined numerically at multiple (e.g., three) specific positions in the graph. The resolution data should consist of at least 12 independent data points collected from a matrix setup (Section 3.1; Kojima design [13], Table S1).

#### 2.5.1 (Statistical) Evaluation

Visual comparison allows identification of potential discrepancies in peak width and peak separation between both instruments. Resolution on the new instrument should be similar or better than on the original instrument.

In numerical evaluation, for the peak width/separation on both instruments to be consistent, the acquired resolution data from the new instrument should be in a range of ±3 SD of the historical resolution data acquired on the original instrument (e.g., control chart data). The comparability of both instruments should be statistically evaluated with a TOST, using a significance threshold of *α* = 0.05.

### 2.6 Peak Area

To evaluate consistency of the relative/total peak areas (i.e., the curve integral) between both instruments, relative/total peak areas of specific peaks or peak groups in the electropherogram should be compared. The peak area data should consist of at least 12 independent data points collected from a matrix setup (Section 3.1; Kojima design [13], Table S1).

#### 2.6.1 Statistical Evaluation

For relative/total peak areas on both instruments to be consistent, the acquired data from the new instrument should be in a range of ±3 SD of the historical data acquired on the original instrument (e.g., control chart data). The comparability of both instruments should be statistically evaluated with a TOST, using a significance threshold of *α* = 0.05.

### 2.7 Measurement Variance

Measurement variance across different instruments, analysts, days, and capillaries/columns indicates whether product analysis has a similar level of precision on both instrument platforms. The measurement variance data should consist of at least 12 independent data points collected from a matrix setup (Section 3.1; Kojima design [13], Table S1).

#### 2.7.1 Statistical Evaluation

For measurement variance on both instrument platforms to be consistent, an SD or RSD analysis may be considered:

SD analysis: The acquired data from the new instrument should have an SD equal or below *x*·SD of the historical data acquired on the original instrument (e.g., control chart data). An empirical starting value *x* would be 2 and depends on the sample size. Non‐inferiority of the SD on the new instrument may be assessed with a one‐sided chi‐square test (*α* = 0.05).

RSD analysis: The non‐inferiority of the RSD may be assessed by a one‐sided chi‐square test (*α* = 0.05), according to which data from the new instrument should be equal or below *y*·RSD of the historical data acquired on the original instrument (e.g., control chart data). An empirical starting value *y* would be 2, typically used in acceptance criteria setting for the intermediate precision parameter of method validations.

In addition, the intermediate precision results of the original method validation may be compared to the results acquired on the new instrument.

## 3 Materials and Methods

### 3.1 Experimental Design and Study Material

**FIGURE 2:**
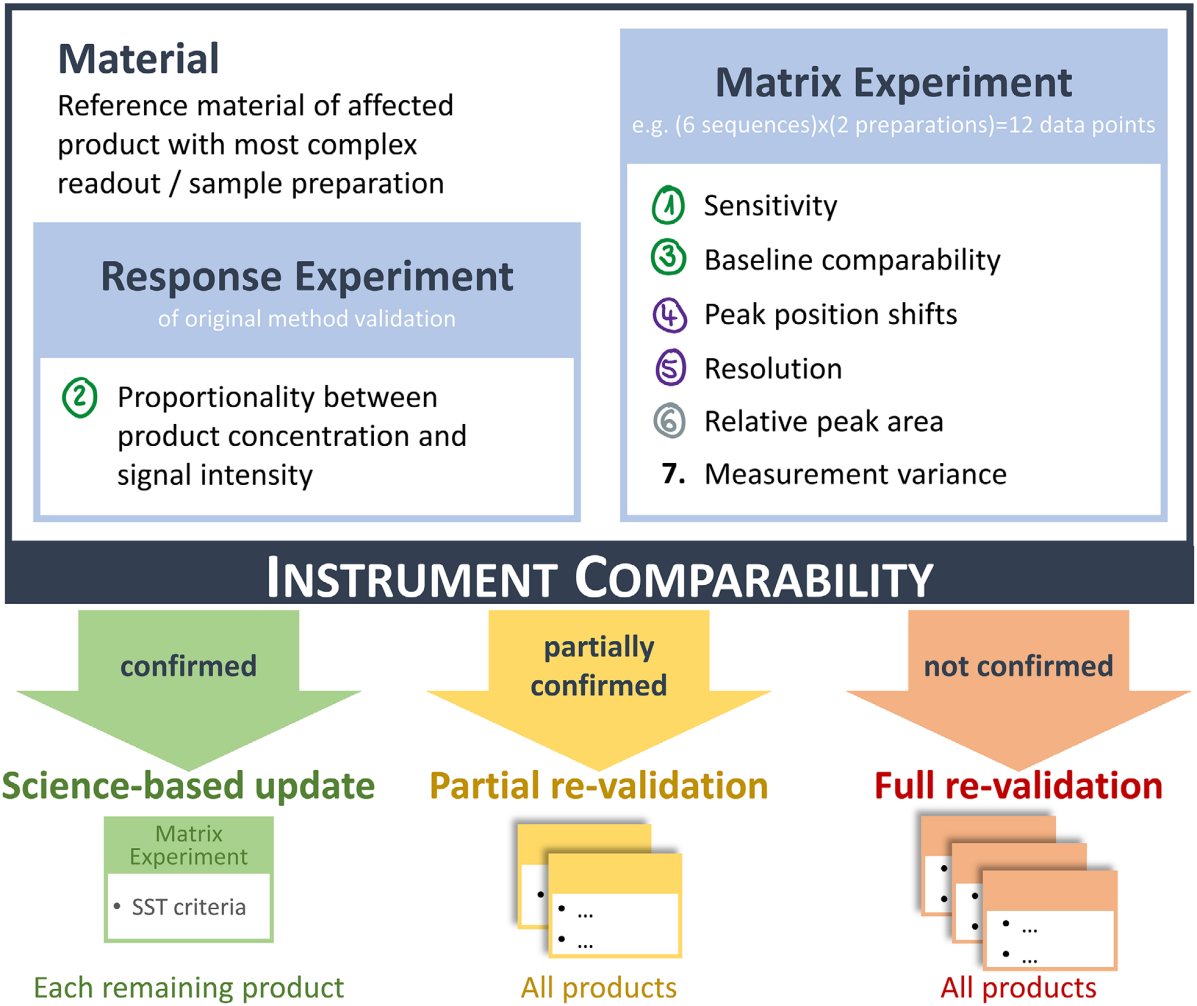
Experimental design of the instrument comparability study: Blue boxes contain experimental setup and derived data of core study. Green, yellow, and red arrows depict possible outcomes and resulting path forward. Below the arrows, the emerging analytical effort for each scenario is illustrated. SST, system suitability test.

The experimental procedure comprises a response and a matrix setup experiment. The parameter “proportionality between product concentration and signal intensity” is derived from the response experiment, while all remaining parameters are evaluated from the matrix experiment (blue boxes in Figure 2).

The sample size of the matrix experiment should at least consist of 12 data points. Other sample sizes may be possible; the design of experiments (DoE) set‐up for the Kojima design should reflect the strongest variance influencing factors. Furthermore, the sample size should be large enough that given the method variability of the new instrument it is possible to reliably pass the TOST. A power analysis should be done with respect to the new instrument and the acceptance criteria from historical data to get an acceptable passing probability (=power of e.g., at least 90%).

To ensure system suitability in all experiments, a product‐independent and well‐studied material is recommended to be used. If available, an instrument specific kit by the manufacturer may be an optimal choice.

If multiple products are affected by the instrument change, it is advisable to use the product with the highest complexity in readout (e.g., close peaks, high number of peaks) and sample preparation (e.g., high number of incubations, buffer exchanges) in the comparability study. Thereby, the study results may be considered indicative also for other affected products/procedures.

Reference material is the preferable material to carry out comparability experiments on the new instrumentation (Figure 2 top left corner), as it is advisable to set acceptance criteria for some parameters from historical data collected on the original instrument. In a GMP environment, such historical data are typically available from control charts containing data of reference material over a long period of time. Control chart data provide high statistical power and a good estimate on expected method variance. Furthermore, reference material is typically generated in high amounts and therefore likely to be available.

In case degraded material adds a significant amount of complexity to the readout, direct comparison of respective profiles from both instruments may be considered to complement the study design.

### 3.2 icIEF Instrument Comparability Study

The study design as described in Sections 2 and 3.1 was applied in an instrument update between two icIEF instruments, ICE3 (original instrument, Protein Simple, Bio‐Techne) and Maurice C (new instrument, Protein Simple, Bio‐Techne). As study material, reference standard material of a globular glycoprotein and the respective icIEF analytical procedure were used. Degraded material of the selected product does not increase complexity of the profile and was therefore not included in the study.

Samples and matrix were prepared immediately before analysis. The matrix, containing urea, methyl cellulose, phosphoric acid as anode spacer, carrier ampholytes, and two *pI* markers, was set up as a master mix and added to each sample of the respective sequence. Samples were detected by UV absorbance at 280 nm. The fluorescence detection mode of Maurice C was not used, as this would be a significant discrepancy from the original analytical procedure validated on ICE3. ICE3 only allows for UV absorbance detection.

Samples were mixed with matrix, pipetted into 96‐well plates for high‐throughput analysis (046‐021, protein‐simple, Bio‐Techne), and injected into Maurice iCIEF Cartridges (PS‐MC02‐C, Protein Simple, Bio‐Techne), set up with catholyte and anolyte solutions by the manufacturer (#102506, Protein Simple, Bio‐Techne).

Peak integration of the product was evaluated in three regions (labeled in Figure 4B), each consisting of a sum of multiple peaks.

System suitability was assessed on Maurice C with the Maurice cIEF System Suitability Kit (#046‐044, Protein Simple, Bio‐Techne) following the manufacturer's instructions and criteria. The peptide panel provided in the kit was analyzed in a bracketing approach at the beginning and end of each sequence. All experiments passed the system suitability test (SST).

Maurice C instruments were operated with the Empower 3 software (Waters). For setup and calibration of Maurice C instruments, 0.5% methyl cellulose (#102730, Protein Simple, Bio‐Techne) and Maurice CIEF Fluorescence Calibration Standard (#046‐025, Protein Simple, Bio‐Techne) were used according to the manufacturer's recommendation.

Collected data were analyzed and integrated in Empower 3 software (Waters). Results were evaluated in Microsoft Office Excel (Version 2308) and Minitab Statistical Software (Minitab 20.2, 64‐bit). For visual evaluation, electropherogram figures of both instruments were generated in Empower 3 (Waters) and overlaid in Microsoft Office Excel.

Acceptance criteria were either visual (baseline comparability, resolution), or adopted from the original method validation on ICE3 (proportionality in response), or calculated from historical measurements (validation or control chart data) on ICE3 (sensitivity, peak positions in *x*, relative peak areas, and measurement variance). Historical ICE3 data were generated in with the iCE CFR software and integrated in Empower 3 (Waters). The applied control chart data were collected from the same reference material over 4 years, on 3 ICE3 instruments, by 7 analysts, using multiple capillaries, and generated a total of 128 data points. Acceptance criteria were identified/calculated as described in Section 2 Theory.

## 4 Results and Discussion

### 4.1 Study Design

The study design allows to generate and evaluate results for systematic instrument comparison and is universally applicable to various methods. In chromatographic and capillary electrophoretic methods, it could be realized following the design outlined in Section 2 and 3.1. Other release analytical methods may need parameter adjustments. Nonetheless, the underlying principle of (a) systematically comparing the method readout and (b) leveraging available historical, large, and thus statistically powerful data, holds potential for all methods.

As path forward resulting from the comparability study, three outcomes are possible:

1. **Instrument comparability can be confirmed across all parameters** (green arrow in Figure 2). The study results may serve as foundation for a **science‐based instrument update**. To expand the study results to further affected products, it is advisable to test each product at least once on the new instrument to avoid unforeseen risks. An efficient approach, conducting a matrix experiment with reference material of each product, using product specific SST criteria as acceptance criteria, would be recommended (green box in Figure 2 and Table S1).
2. **Comparability between the two instruments can only partially be confirmed** (yellow arrow in Figure 2). In a **partial re‐validation,** targeted experiments need to be conducted to address the discovered discrepancies and complement the original method validation for application on the new instrument.
3. **Comparability between the two instruments cannot be confirmed** (red arrow in Figure 2). A **full re‐validation** is required.

### 4.2 icIEF Instrument Comparability Study

**FIGURE 3:**
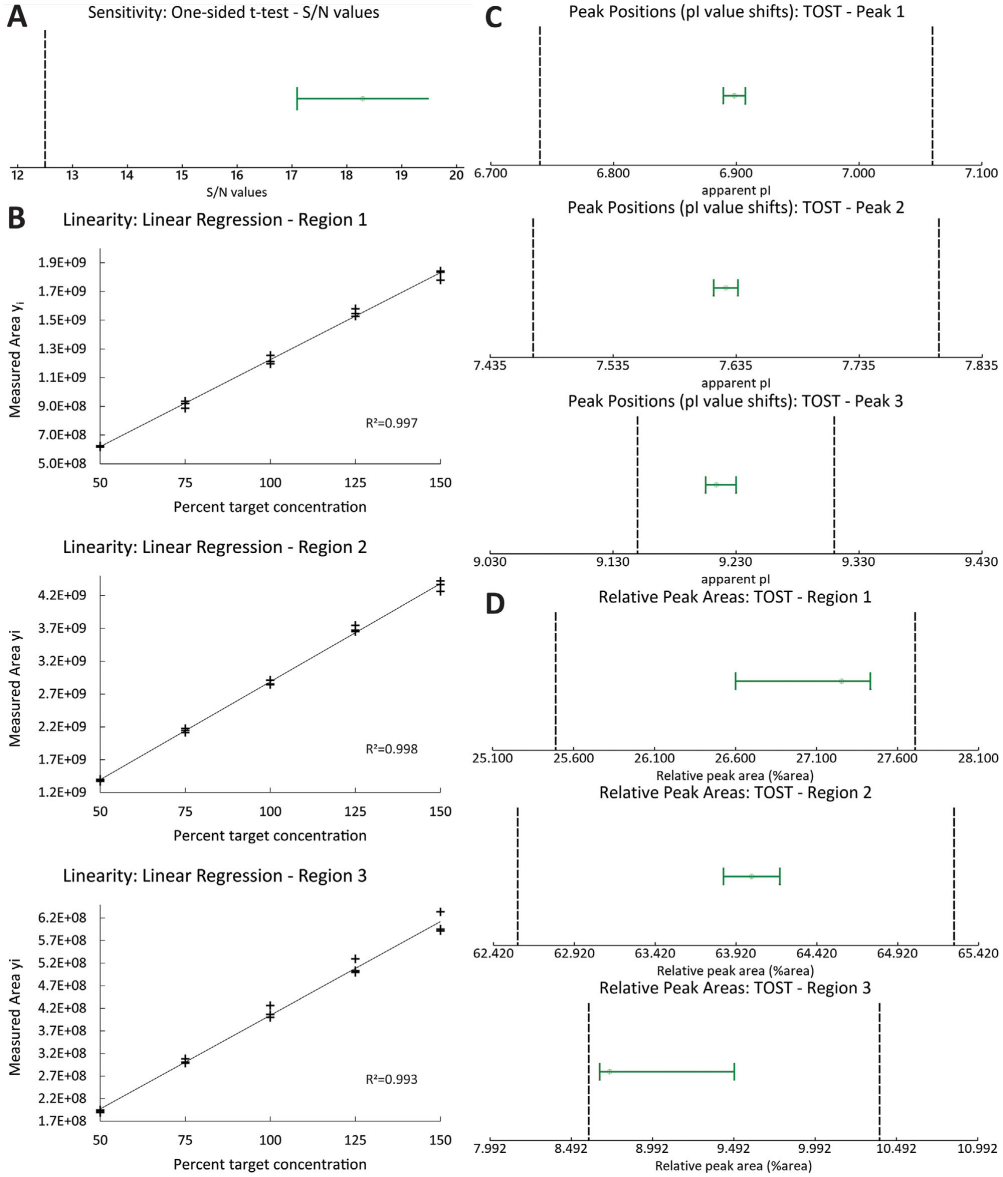
Statistical evaluation of icIEF instrument comparability study results. (A) Sensitivity: *t*‐test of S/N values with a 95% confidence interval (green interval bar) and the lower limit (black dashed line, S/N = 12.5) received from original method validation on ICE3, (B) Linearity: Linear regression plots of measured peak areas plotted against percent target concentration. One subpanel per evaluated region (1, 2, and 3) (C) Peak position shifts in *x*: TOST of apparent *pI* values with a 95% confidence interval for equivalence (green interval bar). Lower and upper equivalence limits (black dashed lines) were calculated from control chart data (mean ± 3 SD range). (D) Relative peak areas: TOST of relative peak areas with a 95% confidence interval for equivalence (green interval bar). Lower and upper equivalence limits (black dashed lines) were calculated from control chart data (mean ± 3 SD range). S/N, signal‐to‐noise; TOST, two one‐sided *t*‐tests.

**FIGURE 4:**
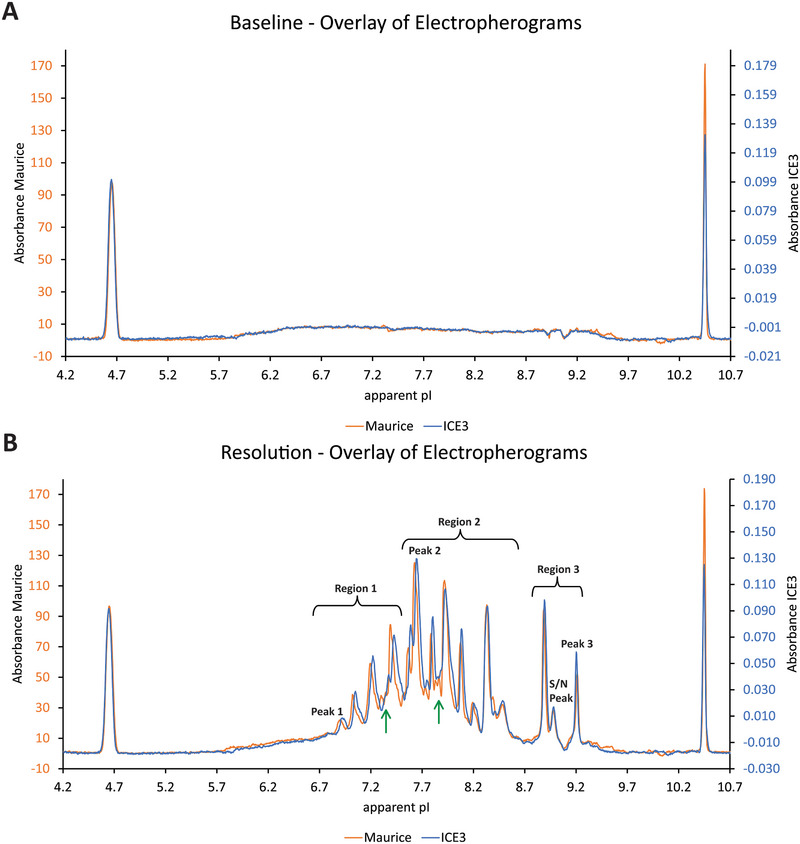
Visual evaluation of icIEF instrument comparability study results. *X*‐axes: Absorbance values in arbitrary units for Maurice C (orange) and ICE3 (blue). *Y*‐axes: Apparent *pI* values. (A) Baseline: Representative overlay of blank electropherograms (orange—Maurice C, blue—ICE3). (B) Resolution: Representative overlay of sample electropherograms (orange—Maurice C, blue—ICE3). Green arrows indicate discrepancies in resolution. Black labels indicate peaks used for evaluation of peak position shifts in *x* and regions used for evaluation of response.

The acquired data in the icIEF instrument comparability study revealed an equal or better performance of Maurice C and met all acceptance criteria set *a priori*. The results are summarized in Figures 3, 4 and Table S2 and explained in more detail in the following paragraphs.

#### 4.2.1 Sensitivity

To investigate sensitivity, the S/N values were determined from the peak used for LOQ determination in the original validation and accordingly collected from the integrals (*n* = 6) of a small peak in the electropherogram at 100% target load (Figure 4). Acceptance criteria for sensitivity were non‐inferiority of the S/N ratio and an equal or lower RSD (%Area) on Maurice C when compared with ICE3. The S/N on Maurice C was non‐inferior to ICE3 data (mean(S/N ICE3) = 12.7), as was confirmed with a one‐sided *t*‐test (95% confidence interval with lower bound 17.1) (Figure 3A). Furthermore, the peak was more robust on Maurice C (RSD (%Area) = 6.2%) than on ICE3 (RSD (%Area) = 9%). Thereby, comparability of the Maurice C instrument with respect to sensitivity was confirmed.

#### 4.2.2 Proportionality Between Product Concentration and Observed Signal

The proportionality was determined according to the procedure of the original method validation in a linearity experiment over a range of 50%–150% target concentration with five equidistant concentration levels and three independent preparations (*n* = 3) per level. Linear regression of product concentration and peak area was determined, and the correlation coefficient calculated for the total peak area (Figure S1) and the individual regions 1, 2, and 3 (Figure 3B, regions of the profile are labeled in Figure 4B). Acceptance criterion for comparability was, according to the original method validation, a correlation coefficient of *r* ≥ 0.98. Values from original validation on ICE3 were 0.990 (Region 1), 0.990 (Region 2), and 0.990 (Region 3); newly measured values on Maurice C were 0.998 (Region 1), 0.999 (Region 2), and 0.996 (Region 3). Thereby, comparability of the Maurice C instrument with respect to the proportionality between product concentration and observed signal was confirmed.

#### 4.2.3 Baseline Comparability

Baseline comparability was assessed visually by overlaying three blank electropherograms of Maurice C measurements with historical ICE3 measurements randomly selected from the control chart set (Figure 4A and Figure S2A,B), assuring variance in instruments, analysts, and reagents. Visual evaluation showed equal occurrence of irregularities in the blank shape (e.g., increasing signal between apparent *pI* 5.7 and 6.5; indentations at apparent *pI* 8.9 and 9.1). Minor discrepancies in the position of irregularities in *x* could be observed (apparent *pI* 9.3–9.5) and were traced back to the usage of different carrier ampholyte batches, which can minimally affect the focusing behavior. No substantial discrepancies or deviating trends were noted on either instrument. Consequently, comparability of the Maurice C instrument with respect to baseline comparability was confirmed.

#### 4.2.4 Peak Position Shifts in *X*

Peak position shifts in *x* were assessed on three peaks located over a range of apparent *pI* 6.5–9.5 in the electropherogram (Figure 4B, black labels). A total of 12 data points (each being a mean value of 6 injections) from the matrix experiment were compared with historical control chart data collected on the ICE3 instrument (128 data points). Results are displayed in Figure 3C. Equivalence of the data was tested using a TOST with a significance level of *α* = 0.05 and could be confirmed for all peaks.

#### 4.2.5 Resolution

Resolution comparability was assessed visually by overlaying three representative electropherograms of Maurice C measurements with historical ICE3 measurements (Figure 4B and Figure S2C,D). Numerical evaluation could not be realized due to a lacking baseline separation between most peaks and a resulting inconsistent resolution calculation between instruments, for example, including or excluding a shoulder peak in peak‐width calculation at half peak‐height. Visual evaluation showed equal overall electropherogram appearance. Individual peak shapes displayed higher resolution on Maurice C (see Figure 4B, green arrows at apparent *pI* 7.2–7.4 or 7.8–8.0), which could either be traced back to the usage of different carrier ampholyte batches or indicated a higher resolution (i.e., better focusing behavior) of Maurice C. Consequently, comparability of the Maurice C instrument with respect to resolution was confirmed.

#### 4.2.6 Relative Peak Area

Relative peak area changes were assessed on three regions of the electropherogram each a sum of multiple peaks. A total of 12 data points (each being a mean value of 6 injections) from the matrix experiment were compared with historical control chart data collected on the ICE3 instrument (128 data points). Results are displayed in Figure 3D. Equivalence of the data was tested using a TOST with a significance level of *α* = 0.05 and could be confirmed for all regions.

#### 4.2.7 Measurement Variance

Measurement variance was assessed on three regions of the electropherogram each a sum of multiple peaks. Data from the matrix experiment comprising 12‐fold sample preparation and measurement by two analysts on two instruments using four different cartridges on 6 days. Acceptance criteria were set by calculating the RSD% values of the relative peak areas of the 3 evaluated peak regions from historical control chart data collected on ICE3 (128 data points) and multiplying by a factor of 2. Resulting acceptance criteria were 95% upper confidence limit of RSD ≤ 2.8% (Region 1), ≤1.4% (Region 2), and ≤6.3% (Region 3).

The RSD on Maurice C was non‐inferior to ICE3 data, as was confirmed with a chi‐square test yielding 95% upper confidence limits at 2.3 (Region 1), 0.9 (Region 2), and 2.4 (Region 3).

Thereby, comparability of the Maurice C instrument with respect to measurement variance was confirmed.

## 5 Concluding Remarks

The presented study design allows for a differentiated analysis of instrument comparability and thus provides a solid scientific foundation for maintaining state‐of‐the‐art analytical instrumentation for release testing. It is a comprehensive approach to rationalize the decision process of how to implement an instrument change in a GMP environment.

The results may either provide a detailed understanding of potential discrepancies between instruments, allowing to rationalize the planning of a partial or full method re‐validation. Or, in case comparability can be confirmed, the data may be a scientific foundation to move analytical procedures seamlessly to the new instrument. In this case, analytical procedures would be maintained and benefit from the advantages of more modern instruments, such as facilitated handling, higher standards in data integrity, or more technical options.

This proposal is structured to be a universal starting point for instrument comparability studies across all analytical methods.

The study design was successfully applied in the GMP environment of QC release analytics. The results demonstrate comparability of analytical results between two icIEF instrument platforms and complement a recent inter‐company study [14] as well as available instrument comparability data by Protein Simple [15]. As described in Section 4.1, to expand the study results to all other affected products, we additionally tested them on the new instrument in a matrix experiment (Figure 2, green box; data not shown), using product specific SST criteria as acceptance criterion. The SST criteria were met in all cases. The results of the comprehensive comparability study together with the passed SST criteria of all affected products present a solid scientific foundation for a seamless continuation of icIEF QC release analytics on the new instrument, Maurice C.

## Author Contributions

**Anne B. Ries**: methodology, validation, formal analysis, investigation, data curation, writing – original draft, writing – review and editing, visualization, project administration. **Maximilian N. Merkel**: methodology, validation, formal analysis, investigation, data curation, writing – review and editing. **Kristina Coßmann**: investigation, data curation. **Marina Paul**: investigation, data curation. **Robin Grunwald**: investigation, data curation. **Daniel Klemmer**: formal analysis, writing – review and editing. **Franziska Hübner**: conceptualization, methodology, resources. **Sabine Eggensperger**: resources, writing – review and editing. **Frederik T. Weiß**: conceptualization, methodology, validation; resources, writing – review and editing, supervision.

## Conflicts of Interest

The authors declare no conflicts of interest.

## Supporting information




**Supporting File 1**: elps70004‐sup‐0001‐SuppMat.docx.

## Data Availability

The data that support the findings of this study are available from the corresponding author upon reasonable request.
