# Supplementary material for: Universal Study Design for Instrument Changes in Pharmaceutical Release Analytics
Source: Electrophoresis. 2025 Jun 25;46(11-12):669–78. doi: 10.1002/elps.70004 (PMC12366236; doi:10.1002/elps.70004)
Supplement: Supplementary file 1 — Supporting File 1: elps70004‐sup‐0001‐SuppMat.docx. [file ELPS-46--s001.docx]

Supporting Information to “Universal Study Design for Instrument Updates in Pharmaceutical Release Analytics”

**Table S1** Matrix setup for measurement variance determination (Kojima design [13])

| **Day** | **Analyst** | **Instrument** | **Capillary** | **No. of sample preparations** |
| --- | --- | --- | --- | --- |
| 1 | A | A | A | 2 |
| 2 | A | B | A | 2 |
| 3 | A | A | B | 2 |
| 4 | B | B | A | 2 |
| 5 | B | A | B | 2 |
| 6 | B | B | B | 2 |


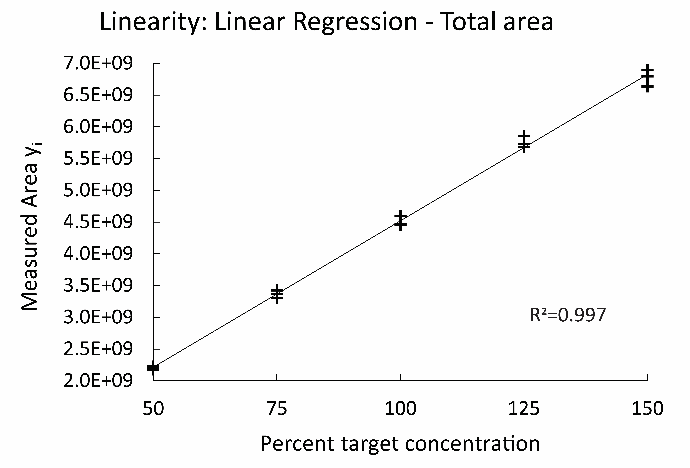


**Figure S1** Linearity: Linear regression plot of total peak area plotted against percent target concentration.

Linearity results: Correlation coefficient of total area from original validation on ICE3 is 0.99, newly measured value on Maurice is 0.999.

**
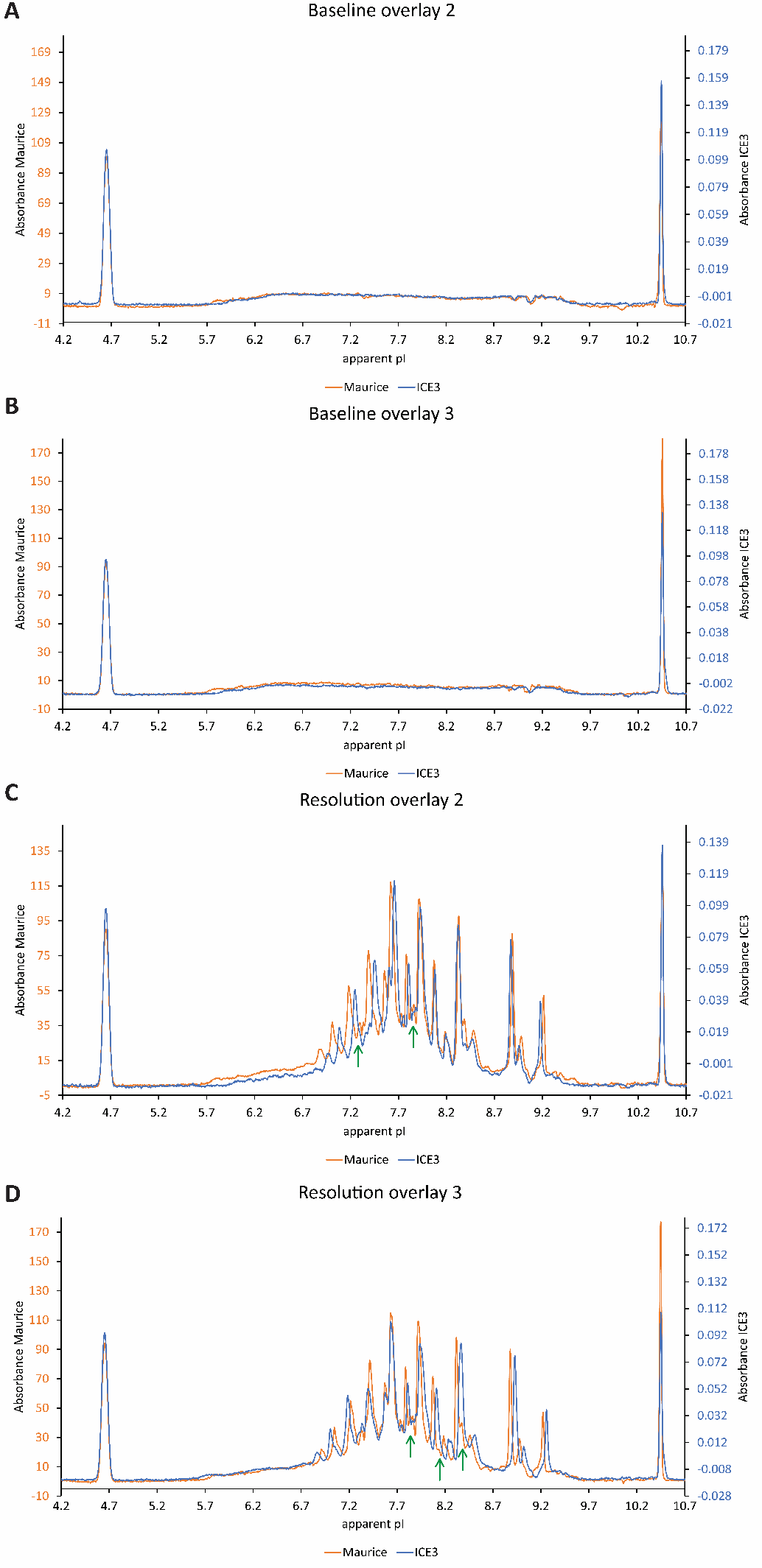
**

**Figure S2** Visual evaluation of icIEF instrument bridging study results. (A) Baseline: Second overlay (orange – Maurice C., blue – ICE3). (B) Baseline: Third overlay (orange – Maurice C., blue – ICE3). (C) Resolution: Second overlay (orange – Maurice C., blue – ICE3). Green arrows indicate discrepancies in resolution. (D) Resolution: Third overlay (orange – Maurice C., blue – ICE3). Green arrows indicate discrepancies in resolution.

**Table S2** Summarized results of icIEF instrument comparability study

| **Parameter** | **Acceptance criteria** | **Historical ICE3 data** | **Maurice C data** |
| --- | --- | --- | --- |
| Sensitivity | S/N: Maurice C ≥ ICE3  RSD(%Area): Maurice C ≤ ICE3 | S/N = 12.7  RSD(%Area) = 9% | S/N 95% lower confidence limit = 17.1  RSD(%Area) = 6.2% |
| Proportionality product conc. / signal intens. | Pearson correlation coefficient of linear fit for all regions / total  r ≥ 0.98 | Region 1: r=0.990  Region 2: r=0.990  Region 3: r=0.990  Total: r=0.99 | Region 1: r=0.998  Region 2: r=0.999  Region 3: r=0.996  Total: r=0.999 |
| Baseline | Visual comparability | See figures 4A, S2A, S2B | See figures 4A, S2A, S2B |
| Peak position shifts in x | 3SD range of historical data  Peak 1: 6.74 to 7.06  Peak 2: 7.47 to 7.80  Peak 3: 9.15 to 9.31 | N/A | 95% confidence intervals for equivalence  Peak 1: 6.89 to 6.91  Peak 2: 7.62 to 7.64  Peak 3: 9.21 to 9.23 |
| Resolution | Visual comparability | See figures 4B, S2C, S2D | See figures 4B, S2C, S2D |
| Peak area changes | 3SD range of historical data  Region 1: 25.49 to 27.71  Region 2: 62.57 to 65.27  Region 3: 8.60 to 10.39 | N/A | 95% confidence intervals for equivalence  Region 1: 26.60 to 27.43  Region 2: 63.84 to 64.19  Region 3: 8.67 to 9.50 |
| Measurement variance | 2xRSD of historical data  Region 1: ≤ 2.8%  Region 2: ≤ 1.4%  Region 3: ≤ 6.3% | N/A | RSD 95% upper confidence limit  Region 1: 2.3%  Region 2: 0.9%  Region 3: 2.4% |
